# The choice between hip prosthetic bearing surfaces in total hip replacement: a protocol for a systematic review and network meta-analysis

**DOI:** 10.1186/s13643-016-0189-5

**Published:** 2016-02-01

**Authors:** Elsa M. R. Marques, Rachel Humphriss, Nicky J. Welton, Julian P. T. Higgins, William Hollingworth, Jose A. Lopez-Lopez, Howard Thom, Linda P. Hunt, Ashley W. Blom, Andrew D. Beswick

**Affiliations:** School of Social and Community Medicine, University of Bristol Canynge Hall, 39 Whatley Road, Bristol, BS8 2PS UK; Musculoskeletal Research Unit, School of Clinical Sciences, University of Bristol Learning & Research Building (Level 1), Southmead Hospital, Bristol, BS10 5NB UK

**Keywords:** Hip prosthesis, Hip implant, Prosthetic implants, Total hip replacement, Metal, Ceramic, Polyethylene, Revision hip replacement

## Abstract

**Background:**

Prosthetic hip implants have many combinations of bearing surface materials, sizes, and fixation techniques, which can determine the quality of life of patients after primary total hip replacement (THR) and the likelihood of needing revision surgery. When an implant fails, patients require revision THR, which is distressing to the patient and expensive for the health care payer. Primary THR is one of the most common elective procedures performed worldwide, with over 300,000 performed annually in the USA and over 80,000 in England and Wales. It is important to review all available randomised controlled trial (RCT) evidence to determine which implant bearing surface materials, size, and fixation technique are most effective for patients.

**Methods/Design:**

This is a protocol for a systematic review and meta-analysis of RCTs comparing outcomes of hip implant bearing surfaces, size, and fixation techniques used in THR. Implant combinations compared in the literature include four bearing surface combinations (metal-on-polyethylene, metal-on-metal, ceramic-on-polyethylene, and ceramic-on-ceramic); two femoral head sizes (large vs small heads); and four fixation techniques (uncemented, cemented, hybrid, and reverse hybrids). The primary outcome will be revision surgery. We will also collect data on patient characteristics, mortality, quality of life, and other outcomes. In network meta-analysis, we will estimate the relative effectiveness of every implant bearing surface, head size (large vs small), and fixation permutation, using evidence where implants have been compared directly in an RCT and indirectly through common comparators in different RCTs.

**Discussion:**

There has been much debate about materials used for prosthetic implants in THR. Different combinations of prosthetic materials, sizes, and fixation, can vary widely in cost and fail at different rates for different patient groups. Given the number of THRs performed yearly, and the increasing use of expensive implants, it is important to review evidence to inform surgeons, patients, and health care providers of optimal implant bearing combinations for given patient characteristics. This review will inform a cost-effectiveness model that will include evidence from other sources, to determine the most effective and cost-effective implant bearing combination for patients.

**Systematic review registration:**

PROSPERO CRD42015019435

## Background

Total hip replacement (THR) is one of the most common elective procedures performed worldwide. In the USA in 2010, the estimated numbers of hospital discharges after THR procedures were 332,000 [1]. In England, Wales, and Northern Ireland, 98,279 THR operations were performed in 2014 [2, 3]. The main indications for elective hip replacement are pain and functional limitations due to osteoarthritis [4, 5].

In a primary THR, both the acetabulum and the femoral head are replaced: a metal stem is inserted into the femur with a modular head, made of metal or ceramic, and articulates with an artificial cup, the acetabular component. The acetabular component can either be a monobloc polyethylene component or a modular component consisting of a metal outer shell with an inner liner made of polyethylene, ceramic, or metal. The femoral stem and the acetabular component are attached to host bone with or without cement. In a resurfacing hip replacement, the femoral head is not removed but is instead trimmed and capped with a smooth metal covering. The acetabulum is removed and replaced with an all-metal monobloc acetabular component.

Implants have four main combinations of head and acetabular materials: metal-on-polyethylene, metal-on-metal, ceramic-on-ceramic, and ceramic-on-polyethylene. Ceramic-on-metal combinations also exist but are extremely rare. Head sizes vary, ranging from 22.225 mm to 50 mm in diameter. Heads are broadly categorised as “large” if 36 mm in diameter and over and “small” if less than 36 mm. As both the femoral and acetabular components can be implanted with or without cement, the possible combinations of fixation are cemented (when both components are cemented), uncemented (when neither prosthesis is cemented), hybrid (when the femoral component but not the acetabular component is cemented) or reverse hybrid (when the acetabular but not femoral component is cemented).

Recent NICE guidance advises the use of prostheses that have rates (or projected rates) of revision of 5 % or less at 10 years [6]. For hip surgeons, implant choice is driven mainly by survival: the implant should outlast the remaining lifetime of the patient and should be easy to revise if it fails. When an implant fails, for example due to infection, dislocation or loosening, patients may endure severe pain and require a revision hip replacement. In England, Wales, and Northern Ireland, in 2014, 10 % (9516) of the THR procedures performed were revision hip replacements [2, 3].

### Why it is important to do this review

There has been much debate about materials used for prosthetic implants in THR. Metal-on-polyethylene cemented implants were developed in the 1950s. They have a long track record of use and are still the cheapest and most prevalent type of hip implant. However, the polyethylene component wears with increased physical activity and load [7, 8]. This results in loosening and bone loss over time, which is of particular significance in younger more active patients. Alternative materials, head sizes, and fixation techniques have subsequently been developed to improve long-term survival and patient outcomes. Metal-on-metal and ceramic-on-ceramic bearings have lower wear rates and larger heads should be more difficult to dislocate but may have higher volumetric wear.

In a study of observational data, metal-on-metal implants failed more often than traditional metal-on-plastic implants [9, 10], which caused much concern to patients and clinicians [11]. Some studies (mainly cohort studies and a few small trials) suggest that newer ceramic-on-ceramic prostheses perform better in younger and active patients [12], and their use is increasing in the UK [13]. However, they can cost up to four times more than metal-on-polyethylene implants. More recently, a systematic review on implants for total and resurfacing hip replacement was published [14]. However, this review was truncated to recent and large studies, focused on functional outcomes, and did not inform the subsequent cost-effectiveness model [15].

We aim to conduct a systematic review of all published randomised controlled trials (RCTs) comparing outcomes of bearing surface combinations, head sizes, and fixation techniques used in THR. We will use network meta-analyses to combine direct and indirect evidence from the RCTs to obtain relative treatment effect estimates of revision rates for bearing surface, head size, and fixation technique permutations available in the literature. Data collected in this systematic review and meta-analyses will inform a future cost-effectiveness decision analytic model to compare the cost-effectiveness of all different implant combinations in current clinical practice.

## Methods/design

### Criteria for considering studies for this review

#### Types of studies

Randomised controlled trials (RCTs).

#### Types of participants

Patients aged 18 years and older receiving primary THR. Populations in trials should have a diagnosis of osteoarthritis in a majority of patients.

#### Types of interventions and comparators

We will include comparisons among all the THR implant combinations listed in Table 1. Although this lists up to 33 different implant combinations, some are rare (e.g. reverse hybrids) and not all implant combinations will be reported in the literature. Eligible studies will make comparisons of any combination of different bearing material combinations, different head sizes (large [≥36 mm] or small [<36 mm]), different fixation techniques (uncemented, cemented, hybrids, or reverse hybrids), or comparisons of any combination of these three aspects with resurfacing.

**Table 1:** All hip prosthetic implant combinations to be compared, if available

Materials	Head size	Fixation technique
Ceramic-on-ceramic	Large ≥36 mm	With cement
	Small <36 mm	Without cement
		Hybrid
		Reverse hybrid
Metal-on-metal	Large ≥36 mm	With cement
	Small <36 mm	Without cement
		Hybrid
		Reverse hybrid
Metal-on-polyethylene	Large ≥36 mm	With cement
	Small <36 mm	Without cement
		Hybrid cement
		Reverse hybrid
Ceramic-on-polyethylene	Large ≥36 mm	With cement
	Small <36 mm	Without cement
		Hybrid cement
		Reverse hybrid
Resurfacings		

Although some materials (e.g. metal-on-metal implants) are now recommended less often in clinical practice, and resurfacing hip replacements are not THR surgeries, they will be included in this review when compared to THR in an RCT, as they may provide important common comparator interventions in the network of studies, facilitating relevant indirect comparisons.

#### Types of outcomes

Implants fail for a myriad of reasons (e.g. dislocation, pain, loosening, infection) [3], and decision to revise a failed implant is always multifactorial. Without one clear reason for implant failure, revision rate and timing of revision is a key proxy measure for effectiveness of implants. Studies which do not report revision, time to revision, or person-years at risk will not be included in the meta-analysis of primary outcome but will be included in the narrative review and analysis of secondary outcomes.

#### Primary outcomes

The primary outcome will be the revision rate for the primary THR surgery at any time after surgery.

#### Secondary outcomes

Secondary outcome data collection will include the following:

- Number of revisions at all follow-up times reported, and Kaplan-Meier curves, to facilitate fitting survival models;
- Mortality and time to death;
- Complications (e.g. infections);
- Generic quality of life scores, such as the EuroQol questionnaire and other preference based utility scores [16];
- Other patient-reported scores. These include, the Oxford Hip Score [17] and Western Ontario and McMaster Universities Arthritis Index [18];
- Surgeon completed scores such as the Harris Hip Score [19];

### Exclusion criteria

We will exclude studies on revision THR; emergency surgery, studies where the majority of patients (*n* > 50 %) are receiving THR for less common causes (e.g. osteonecrosis); and comparison of single components (e.g. stems, cups, shells) when they do not produce a different bearing surface combination on their own. We will also exclude studies that only report outcomes gained from revised implants or autopsies, and laboratory and animal studies.

### Search methods for identification of studies

Our literature search will target studies comparing different bearing surface materials and different head sizes. The literature on fixation technique is extensive, and a systematic review was published in 2013 [20]. We will extract data from the RCTs included in their review and update their search to identify more recent RCTs.

#### Electronic searches

The following electronic databases will be searched, without language restrictions:

- MEDLINE;
- Embase;
- Cochrane Library (including reviews, trials, technology assessments, economic evaluations, and Cochrane groups);
- Trials databases— clinicaltrials.gov, World Health Organization International Clinical Trials Registry Platform, EU Clinical Trials Register.

The search strategies are based on those commonly used in Cochrane reviews, developed by the authors in collaboration with three orthopaedic surgeons and an information specialist. Search strategies are customised for each database. A generic search strategy is shown in Table 2.

**Table 2:** Search strategy for Embase

1. hip prosthesis.mp. or exp hip prosthesis/
2. hip prosthesis/ or total hip prosthesis/ or exp hip arthroplasty/ or total hip.mp.
3. exp hip surgery/ or hip/
4. hip replacement.mp
5. (THR or THA).tw.
6. (hip adj3 (arthropl$ or pros$ or surg$ or replac$)).ti,ab.
7. Hip joint.mp
8. Or/1-7
9. randomised controlled trial/
10. “randomised controlled trial (topic)”/
11. controlled clinical trial/
12. randomi#ed.ab.
13. randomly.ab,ti
14. random.ti
15. random*.tw
16. “clinical trial (topic)”/
17. trial.ab,ti
18. meta-analys:.mp
19. Systematic review.tw.
20. Search:.tw
21. Review.pt.
22. Or/9-21
23. ceramic.mp. or Ceramics/
24. Zirconium.mp. or Zirconium/
25. alumina.mp. or Aluminum Oxide/
26. oxinium.tw
27. cerasul.tw.
28. CoM.tw.
29. CoC.tw.
30. CoP.tw.
31. polyethylene.mp. or Polyethylene/
32. plastic.mp. or Plastic/
33. UHMWPE.mp. or ultra high molecular weight polyethylene/
34. XLPE.mp.
35. X3.mp.
36. Metal on metal joint prosthesis/ or metal.mp. or metal/ or metal implant/
37. MoM.mp.
38. cobalt.mp. or Cobalt/
39. chrome.mp or chromium/
40. metallic.mp.
41. metasul.tw.
42. MoP.mp.
43. hard on hard.mp.
44. Hard-on-soft.mp.
45. alloy.mp. or alloy/
46. bearing.mp
47. (resurf$ or re-surf$).mp. [mp = ti, ab, rw, sh]
48. 22 mm.tw or 22 mm.tw
49. 24 mm.tw or 24 mm.tw
50. 26 mm.tw or 26 mm.tw
51. 28 mm.tw or 28 mm.tw
52. 30 mm.tw or 30 mm.tw
53. 32 mm.tw or 32 mm.tw
54. 34 mm.tw or 34 mm.tw
55. 36 mm.tw or 36 mm.tw
56. 38 mm.tw or 38 mm.tw
57. 40 mm.tw or 40 mm.tw
58. 42 mm.tw or 42 mm.tw
59. 44 mm.tw or 44 mm.tw
60. 46 mm.tw or 46 mm.tw
61. 48 mm.tw or 48 mm.tw
62. 50 mm.tw or 50 mm.tw
63. 52 mm.tw or 52 mm.tw
64. 54 mm.tw or 54 mm.tw
65. 56 mm.tw or 56 mm.tw
66. 58 mm.tw or 58 mm.tw
67. 60 mm.tw or 60 mm.tw
68. (BHR or Conserve Plus or Durom or Cormet or ASR or ReCap).ti,ab
69. head size.mp.
70. femoral adj2 size
71. large adj2 head
72. femoral adj2 head
73. or/23-72
74. 8 and 22 and 73

#### Searching other resources

We will inspect reference lists of published papers and citations of key articles using Web of Science citation tracking, to identify further studies. Websites of orthopaedic conferences since January 2012 will be examined to identify studies which have been presented but not yet fully published. Potentially eligible references identified will be retrieved.

### Data collection

#### Selection of studies

All titles and abstracts will be screened independently by two reviewers. Both reviewers will independently decide on whether to obtain the full papers for further assessment or exclude the study. Full text papers will be obtained for all potentially relevant studies and independently examined by both reviewers to assess final inclusion or exclusion. At both stages, disagreements will be resolved by consensus, with input from other team members if necessary.

Where possible, studies will be identified by trial registry numbers to flag multiple publications from the same RCT. Where trial registry numbers are not available, we will identify multiple publications from RCTs by examining information on authors, region or centre, participant numbers, calendar years of recruitment, interventions compared, study title, or by contacting the authors.

Data from multiple publications on the same RCT will be combined into a single data entry.

#### Data extraction and management

Data extraction will be carried out by one reviewer and checked by the other for accuracy. We will extract data on the following:

- Participant details, such as eligibility criteria, proportion of patients with osteoarthritis (if available), number of participants, age, and gender;
- Details on the intervention, such as components, materials, fixation technique, head size, and manufacturer;
- Number of revision surgeries and outcomes defined above;
- Trial details, such as country of origin, authors, study year, and years of patient recruitment;
- Surgical details, such as surgical approach;
- Resource use data (if available), such as operation time, length of hospital stay, and follow-up visits.

We will collect number of events (e.g. number of revisions) and dichotomous outcomes and means, medians, and measures of variance (i.e. standard deviations, confidence intervals, interquartile ranges, and ranges) for continuous outcomes.

Data will be entered into an Access Database designed for this study.

#### Assessment of risk of bias in included studies

We will use the Cochrane Collaboration risk of bias tool to assess selection, performance, detection, attrition, and reporting bias [21]. An attempt will be made to detect selective outcome reporting by comparing registered protocols to published outcomes. We will report summary assessments of risk of bias (high, low, or unclear) for each outcome in each trial.

#### Measures of treatment effect

Where possible, we will extract data according to the intervention patients were randomised to, in line with intention to treat principles. The measure of treatment effect for revision surgery, the primary outcome, will be the hazard ratio. For secondary outcomes, such as patient-reported outcome scales, will use mean differences or standardised mean differences as measures of treatment effect, depending on whether the same scale is used or not.

#### Unit of analysis issues

The unit of analysis will be the patient hip, with a single measurement for each outcome from each patient hip. Patients who have bilateral THR will be analysed as a single measurement of time to first revision.

#### Dealing with missing data

We will contact authors for additional information when necessary. This may include missing data, loss to follow-up, and unpublished outcomes, as well as study characteristics and issues relating to risk of bias. We will also contact authors of registered trials that have not published their results. A maximum of three attempts will be made to contact authors, by e-mail initially, and/or telephone later.

#### Assessment of reporting biases

If 10 or more studies are included in the pairwise meta-analyses, we will also produce funnel plots. If asymmetry is found, we will investigate potential causes. Heterogeneity between studies can lead to asymmetry, without reporting bias present [22]. We will look at the spread of across studies in the analysis (e.g. size, setting) and study characteristics (e.g. differences in implants compared, populations, baseline characteristics) to determine whether asymmetry may be due to publication bias or genuine differences between studies. Inspection of trial registries will identify trials with delayed reporting.

This protocol follows the PRISMA-P 2015 guidelines [23].

### Data synthesis

#### Descriptive analyses

We will summarise key findings and the report of other outcomes descriptively.

The validity of the data synthesis will depend on the assumption that included studies do not differ in important effect modifiers [24–26]. Clinicians identified patient age and surgical approach as important effect modifiers. We will further explore whether other patient characteristics (e.g. baseline descriptive variables such as gender, weight) are balanced across trials and the network of studies and seek clinical advice to assess the plausibility of this assumption. Imbalances in these characteristics may explain potential heterogeneity or inconsistencies across trials. If enough data are available, we will control for effect modifiers using meta-regression.

For the primary outcome, we will use hazard ratios to compare revision rates for different interventions. In the main analysis, we will assume constant hazards with an adjustment for early revisions (due to causes including infection or surgical errors) and use the longest available follow-up period. In a second analysis, we will assume piecewise constant hazards considering three follow-up points (an “early stage” of 1 or 2 years after primary surgery, a “medium stage” possibly 5 years, and a “late stage” ideally at 10 years), which allows for time-varying deviations from the proportional hazards assumption. For both analyses, we will estimate person-years at risk from the mean follow-up times reported in the studies, or use median follow-up or fixed follow-up if the former is not available.

#### Pairwise meta-analysis

Statistical integration will start with standard meta-analysis for each pairwise comparison of prosthesis combination and year of outcome reported. We will explore heterogeneity including risk of bias and other potential effect modifiers (more information in heterogeneity assessment section). We will report both fixed-effect and random-effects meta-analysis results and quantify between-study heterogeneity using the between-study variance (*τ*^2^) and the *I*^2^ statistic [27].

#### Network meta-analysis (NMA)

We will construct a network of studies comparing implant combinations. NMA allows the synthesis of results from trials of interventions that form a connected network of intervention comparisons, so that direct (head-to-head) and indirect (through common comparators in different RCTs) evidence can be statistically combined [24, 28, 29]. NMA also enables the ranking of treatments according to the probability that each is the best, second best, and so on, for a given outcome.

NMA assumes consistency between direct and indirect evidence for a given contrast [30]. NMA can only be conducted on evidence networks that are connected. The primary analysis will be the most disaggregate level of implant combinations that data allow. In the most aggregate case, we will compare implants by bearing surface combination only. For a more refined analysis, we will expand the network to include implant combinations by head size and fixation technique. This will mean more implant combinations or “nodes” being compared in the network. The network will also include studies that compare primary THR with resurfacing hip replacement to provide further indirect evidence. All analyses will be performed within a Bayesian framework, evaluated using Monte Carlo Markov chain simulation computed in WinBUGS.

NMA methods have been extended to estimate treatment effects with multiple follow-up times given assumptions about the underlying time-course of the treatment effects [31, 32]. We will fit survival curves with hazard ratios that may vary with time (e.g. piecewise constant). This information will define a distribution of effects across time and inform the extrapolation of long-term outcomes from other data sources to inform the future economic model.

#### Assessment of statistical inconsistency

The statistical agreement (often called consistency or coherence) between the various sources of evidence in the networks of interventions will be evaluated. In NMA, we require that the relative effects on an appropriate scale (log-hazard here) “add-up”, in the sense that the log-hazard ratio for the comparison of implants A vs C is the sum of the log-hazard ratio for implants A vs B and B vs C. We will implement both a local approach for comparisons involved in a closed loop of evidence (using node splitting [25, 30]) and a global approach to examine evidence of inconsistency in the network as a whole. Results will be presented separately for direct comparisons, indirect comparisons, and network meta-analyses. This statistical assessment of consistency complements our clinically focussed evaluation of the plausibility of consistency through the assessment of clinical homogeneity of patient characteristics in the included studies.

#### Subgroup and sensitivity analyses

These will examine the extent to which study characteristics explain between-study heterogeneity [33]. If data allow, we will investigate whether revision rates vary according to key participant and trial characteristics, including age and surgical approach, in meta-regression analyses.

If a majority of studies (>50 %) are assessed as being at low or unclear risk of bias and these form a connected network of all evaluated implant combinations, our primary analysis will include only these RCTs, with the full network of studies results as secondary analysis. In sensitivity analysis, we will then re-estimate the results also including studies with potential high risk of bias. We will perform sensitivity analysis to the model assumptions, particularly to the model for the survival curve and hazard ratio (i.e. assuming increasing hazards, constant hazards, etc.)

#### Secondary analyses

If data allows for meta-analysis of secondary continuous outcomes, such as patient-reported outcome scales, we will use mean differences, or standardised mean differences, depending on whether the same scale is used or not, to measure similar outcomes across studies.

## Discussion

There has been much debate about materials used for prosthetic implants in THR. Different combinations of prosthetic materials, sizes, and fixation can vary widely in cost and fail at different rates for different patient groups. Given the number of THR surgeries performed yearly, and the increasing use of expensive implants, it is important to review all available evidence to inform surgeons, patients, and health care providers of optimal implant combinations for given patient characteristics.

A recent health technology assessment report reviewed the clinical effectiveness and cost-effectiveness of hip resurfacing and THR [14]. However, the outcomes reported focused on surgeon and patient function scores, and only large studies (with sample size larger than 100 patients) published between the years of 2008 and 2013 were included. The subsequent economic model included only five types of commonly used prosthesis, did not take into consideration head size, and the model parameters were not populated by the systematic review evidence.

This review will compare the evidence on implant bearing surfaces, head sizes, and fixation techniques that are used in primary THR. This review will synthesize all direct and indirect evidence available in the literature to determine the most effective implant type for a patient group of given characteristics. Our analyses will be intended to inform health care decisions in the long term, which may entail extrapolation of results from studies where only short-term follow-up periods were considered. The potential problems of such extrapolations have been highlighted before in the field of total hip replacement [34], and therefore, we will interpret these results cautiously. The results of this review will be combined with other data sources to inform a cost-effectiveness model to determine the most cost-effective implant combination for each patient group.

## Abbreviations

NICE: National Institute for Health and Care Excellence
NMA: network meta-analysis
PRISMA-P 2015: Preferred Reporting Items for Systematic Review and Meta-Analysis Protocols 2015 statement
RCTs: randomised controlled trials
THR: total hip replacement
WinBUGS: Windows-based version of the Bayesian inference Using Gibbs Sampling software
